# Market Opportunities for Hay Milk: Factors Influencing Perceptions among Italian Consumers

**DOI:** 10.3390/ani11020431

**Published:** 2021-02-07

**Authors:** Nadia Palmieri, Alessandra Pesce, Milena Verrascina, Maria Angela Perito

**Affiliations:** 1 CREA–Research Center for Engineering and Agro-Food Processing, 00015 Monterotondo, Italy; 2 CREA, Council for Agricultural Research and Agricultural Economics Analysis Research Center for Politics and Bioeconomy, 00198 Rome, Italy; alessandra.pesce@crea.gov.it (A.P.); milena.verrascina@crea.gov.it (M.V.); 3 Faculty of Bioscience and Technology for Food, Agriculture and Environment, University of Teramo, 64100 Teramo, Italy; maperito@unite.it; 4 UR ALISS, INRAE, Université Paris-Saclay, 94205 Ivry-sur-Seine, France

**Keywords:** consumer attitudes, willingness to consume, hay milk, geographical indication, traditional food

## Abstract

**Simple Summary:**

Consumer’s awareness of milk quality has been largely increased by real food safety scares, environmental issues, and the effects of food on health. Consumers are asking about how the cows were fed and treated. In this framework, despite their appearing to be copious research in the current literature on milk, the consumer acceptance of hay milk in Italy is largely unexplored. Using a structured questionnaire, we found that aspects such as curiosity, the production place of food, the local culinary traditions, label, and environmental issues play an important role in the people’s consumption intentions for milk from hay-fed cows. This study could provide useful implications for food manufacturers and facilitate the design of marketing strategies for hay milk produced in the Centre and South of Italy.

**Abstract:**

Central and South Italy are characterized by small-scale dairy farms and growing abandonment by farmers for other, better-paid, off-farm jobs. New marketing concepts for milk can be one solution to remunerate mountain farmers for their efforts. This study investigates the potential market for hay milk in Italy. In particular, we want to understand which variables drive the people’s willingness to consume hay milk, and if the European food quality certification schemes impact on people’s willingness to consume milk from hay-fed cows. Data were collected from a sample of consumers from Central and South Italy (*n* = 331) using a web-based survey. Later, a discrete choice probit model was applied. The main results indicate that aspects as curiosity, the production place of food, the local culinary traditions, label, and environmental issues play an important role in the people’s consumption intentions for milk from hay-fed cows. Moreover, the survey highlights that the respondents had positive opinion towards hay milk and highlighted some important marketing implications for the Italian milk sector. The study findings could encourage discussion about a niche market to boost local growth, initiating a process of improving livelihoods, certification of products, and use of the marketing tools addressed towards a specific milk consumer’s profile. In other words, the study could provide useful implications for food manufacturers and facilitate the design of marketing strategies for hay milk produced in Central and South Italy.

## 1. Introduction

Consumer’s awareness of milk quality has been largely increased by real food safety scares [1,2], environmental issues [3,4], and the effects of food on health [5]. Unhealthy food choices become a threat to the consumer lifestyle [6]. 

In this context, consumers are asking about how the cows were fed and treated (growth hormones and/or antibiotics, etc.) [7]. In the current marketplace, labels exist for nearly every question and companies answer consumers’ fear with different food certifications to confirm the conformity of certain products to a set of standards. In fact, consumers are informed of the differences between ordinary and green milk in terms of safety, geographical origin, environmentally friendly production, and animal welfare with several recognizable food logos. 

Labels are most commonly recognized as tools to aid in the consumers’ decision-making process, even if there exist a wide variety of labels on the market, which sometimes provide redundant information [8]. The information provided by labeling give the consumer the opportunity to make more informed choices and take into account more complex aspects of consumption, ones that are not directly verifiable by the consumer either before or after purchase [9]. These include the environmental, social, and ethical features of the product. 

However, whether consumers gain from being provided with additional information depends on how receptive they are to the messages [10]. Labels typically emphasize process over product and allow companies to signal quality and the presence of specific attributes to build the potential for a price premium [11,12,13]. 

In particular, in the last few years, consumers are more and more interested in organic, traditional, and local production [14]. Considering traditional and local food products, consumers perceive these foods as having a strong distinctive character linked to the cultural heritage [15,16]. There is plenty of evidence in the literature that the local and traditional labels affect consumers’ food choices and that they are more willing to buy food products originating in some specific area [17,18,19,20,21]. Moreover, consumer interest in organic foods has exhibited continued growth for the past few decades. According to some authors [21,22], organic consumers put more emphasis on the origin of the products than other quality cues. 

The production method is a differentiating characteristic of organic foods and past studies have found that demand is mainly driven by consumers’ environmental concerns [23,24] and more private concerns such as health and food safety [25,26,27]. This growing interest creates a demand for food products with specific characteristics, particularly those that are linked to their geographical origin and their production method [27]. 

Previous empirical studies suggest that most consumers are aware of the milk origin and tend to choose green labeled if they are less expensive than ordinary ones [28]. For this reason, dairy producers have recently started to explore other labels [29]. One of these is hay milk, a new niche market with a completely new logo. 

In this framework, despite their appearing to be copious research in the current literature (see, e.g., in [30,31,32]) on milk, the consumer acceptance of hay milk in Italy is largely unexplored. To the best of our knowledge, this is the second paper dealing with consumer acceptance of hay milk in Italy (the pioneering paper was that of Busch et al. [33] considering the hay milk from South Tyrol—a province of Northern Italy). To our best knowledge, no studies have been conducted to evaluate the acceptance of milk from hay-fed cows produced in other regions of Italy. This is the first paper investigating the issue considering the hay milk from Central and Southern Italy, the most important individual factors, and how the European food quality certification schemes shaping the probability of acceptance on the part of consumers.

This paper tries to fill a gap in the literature, with the purpose of understanding the Central and Southern Italian consumers’ willingness to consume milk from hay-fed cows. Smallholder dairy development is a powerful tool to boost local growth, initiating a process of improving livelihoods, certification of products, and use of the marketing tools.

In particular, we want to understand which variables drive the people’s willingness to consume hay milk, and if the European food quality certification schemes impact on people’s willingness to consume milk from hay-fed cows. 

The paper is structured as follows. Section 2 provides a brief background on both the European food quality certification schemes and milk. Section 3 describes the materials and methods used. The results are presented in Section 4 and are discussed in Section 5. In addition, Section 6 concludes with some considerations.

## 2. Background

Italy is the European country with the highest number of food products with a designation of origin and geographical indication recognized by the European Union. The EU’s system of geographical indications aims to protect the economy of the territory and boost the resilience of localized agri-food systems and wider processes of rural development [34,35].

The indissoluble link with the territory of origin supports the social cohesion of the entire community [36] and preservation of ecosystems. Currently, the legislation system is subject to EC regulations (n. 510/2006 on protected designation of origin (PDO) and protected geographical indication (PGI) [37] and 509/2006 on traditional specialty guaranteed (TSG) [38]). The PDO, PGI, and TSG schemes [39] were introduced not only as a way to support consumers’ decisions, but also as a means of food and safety control [27,40,41]. 

In particular, TSG covers agricultural products and foodstuffs that are produced using traditional raw material or traditional production methods, or that have a traditional composition, with no restriction as to the product’s geographical origin. Moreover, operators who intend to produce, process, pack, and market TSG-labeled hay milk must also comply with and accept the Commission Regulation (EU) 2016/304. 

The hay milk with the TSG can only be applied to milk obtained from cows fed with at least 75% hay (supplemented with small amounts of bran and protein plants) in winter, and fresh grass (herbaceous plants) in summer. According to the Italian Minister of Agriculture, production is still not widespread in Italy (farming hay milk is a rarity) despite the fact that this product can guarantee development in mountain areas. The areas of greatest milk production with hay milk certification are mainly in Northern Italy near the Alpine production areas. 

However, Southern Italy is strongly characterized by very small municipalities and significant depopulation rates, which base their local economy on small-scale farms. Often the production uses sustainable methods and farming practices (for example, extensive livestock is the method widely utilized in mountain areas). If we consider the mountain areas economic recovery, such as the areas affected by the earthquakes in Southern and Central Italy (L’Aquila in 2009, Amatrice in 2016, etc.) and other natural disasters, hay milk could be the way to restart the livestock sector and the local economy. In these southern areas of Italy, dairy is considered a commodity and the price of milk is so low that it does not allow real remuneration for farmers. 

According to the work in [42], the price difference between conventional milk and hay milk is quite remarkable. For example, in Austria, in 2018, the price of conventional milk was 36.84 euros per 100 kg, against 43.7 euros/100 kg for hay milk. Moreover, organic milk was paid at 50.5 euros/100 kg, while that of organic hay milk at 55.3 euros per 100 kg.

In this contest, labeled hay milk could be able to restore a qualitative and economic value to milk that respects a high-level standard of quality [33]. From this point of view, in mountain areas, hay milk could represent a key element to support the local economy and, at the same time, contribute to preserving biodiversity, the landscape, and to contain depopulation. However, hay milk could represent a real opportunity for marginal areas on condition of knowing the real preferences and willingness to purchase of consumers.

## 3. Materials and Methods

### 3.1. Conceptual Framework

**Figure 1:**
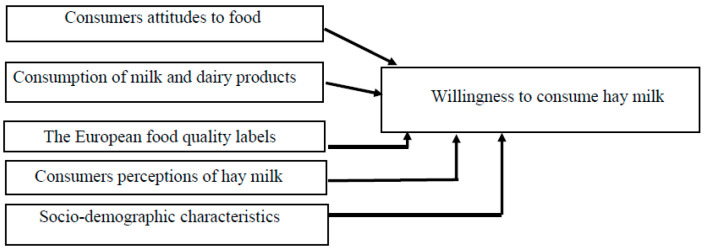
Conceptual framework.

Following Verbeke et al. [43], the study applied a research framework with consumers’ use of the European food quality certification schemes as the behavioral response to willingness to consume hay milk (Figure 1). The baseline assumption is that consumers’ attitudes to food, consumption of milk and dairy products, the European food quality labels, consumers’ perceptions of hay milk, and socio-demographic characteristics of sample have an impact on consumers’ behavior. In particular, the framework of the study is based on a classical model of consumer decision-making [43,44] in which consumers attitudes to food, consumption of milk and dairy products, the European food quality certification schemes, consumers perceptions of hay milk as well as socio-demographic characteristics of sample are hypothesized to drive attitude formation and a subsequent behavioral response (i.e., the willingness to consume hay milk in this study).

### 3.2. Data Collection and Sample

The people’s consumption intentions for hay milk were studied using a structured online survey that was developed for this purpose. In keeping with some studies [45,46,47] about consumer behavior, people were recruited through invitations to participate in the online survey (using the Google Drive platform) via social networks. Moreover, following some authors [25] a snowball sampling recruitment was also adopted, using the emails of the authors’ interpersonal relations to reach a large number of respondents. Given the recruitment method used, the sample cannot be considered representative of the entire Italian population as happens in many studies about consumer behavior [25,33,47,48]. Moreover, a pre-test was conducted on 50 consumers.

Respondents were recruited in Central and Southern Italy. The initial sample was composed of 350 people; 19 respondents were eliminated because they were not milk consumers. The final sample was 331 people and data were collected between January and July 2020. 

### 3.3. Questionnaire

Following the conceptual framework (Figure 1), the questionnaire was composed of 8 pages, in 5 sections, and took participants approximately 10 min to complete.

It is important underlines that the study did not require ethics committee approval for their survey. The research followed the Italian National law (d.lgs. 196/2003) and following modifications by the EU Regulation, prior to answering the questions, participants were briefly informed by research staff about the project that motivated the survey and their free decisions on their involvement on the research and ensuring them that there was no explicit or implicit coercion. Moreover, all information provided for the study is treated confidentially and the respondents’ identities were anonymous. All participants gave their informed consent before answering the questionnaire.

The first section was titled “Consumers attitudes to food” with the aim to focus on both respondents’ food habits (i.e., omnivore, vegan, or vegetarian) [45] and how general food aspects influence respondents’ choices in terms of nutrition and energy (*nutritional aspects, energetic aspects*), people’s attention to both origin of raw materials (*origin_material_raw*), production place of food (*prod_place*), and local culinary tradition (*local_tradition*) [25,45,49,50].

The second part of the questionnaire was titled “Consumption of milk and dairy products” and it investigated the frequency of milk consumption and the type of milk preferred [49], including the agreement/disagreement with some statements such as milk being a fundamental food in a diet (*fundamental_food*), the milk’s taste being an important aspect to consider (*taste*) or there being no differences between them (*no_difference*) [33]. Furthermore, respondents’ consumption of dairy products (*dairy_products*) was investigated [51,52].

In the third section of the questionnaire, titled “the European food quality labels”, participants were questioned on their ideas about the European food quality certification schemes [33,49,50]. In particular, they were asked their agreement/disagreement with some statements about organic food, such as organic food being less impactful than conventional food (*organic_less_impact*), and whether it is safer than conventional food (*organic_safer*) [33,49]. Moreover, the respondents’ ideas about geographical indications of food (i.e., PDO, PGI, and TSG quality labels) were investigated in terms of their agreement/disagreement with some statements such as PDO products characteristics being due to the production place (*pdo_only_land*); the quality of PDO food being better than conventional food (*pdo_better_conventional*); and PGI certified food being produced in only a particular geographical area (*pgi_geogr_area*) [50,53]. Moreover, the participants ideas about food with traditional specialty guaranteed (TSG) certification were also investigated, including questions about the social, economic, and environmental implication of this certification [33,53].

In the fourth part of the questionnaire, called “Consumers perceptions of hay milk”, participants’ ideas about hay milk were investigated [33]. In particular, their familiarity (*hay_milk*) with this kind of milk, their past consumption of hay milk (if any) (*hay_milk_past*) [45], and their willingness to consume hay milk (*hay_willing*) were investigated using a binary choice (Yes or No) [33]. Following the work in [33], the participants were asked about what they associated with the term hay milk to understand if people knew the definition of hay milk in terms of feed used (*hay_assoc*) and livestock management applied (*hay_milk_livestock*). Finally, the people’s motivations to consume hay milk were measured by asking a number of questions related to taste, curiosity (*hay_milk_curiosity*), nutritional aspects, and hygienic issues, as well as socio-economic aspects and environmental ones (*hay_milk_less_env_impact*) [33].

It is important to underlines that for the questions in sections 1–4, people were asked to answer on a 10-point Likert scale (1 = disagree completely, 10 = agree completely) [47] except for some questions (i.e., *hay_milk*, *hay_milk_past*, and *hay_willing*) where a binary choice (Yes or No) was applied. 

The questionnaire ended with socio-demographic questions related to the respondents’ sex (*gender*), age, and education level. It is important underlines that due to the high percentage of refusal to answer a direct question about family income during the pre-test survey [54]; this question was excluded in the final version of the questionnaire.

### 3.4. Data Analysis 

The study aimed to identify factors affecting the decision of the people to consume hay milk. Given the dichotomous nature of the consumers’ answers, a qualitative response model was appropriate [55]. It is important to underline that qualitative response models are often useful when assessing consumer characteristics that are associated with consumption decisions [55]. In our case, in order to lead an analysis of the consumers’ behavior about hay milk preferences, a discrete choice probit model for binary choice (Yes or No) responses to the hay milk consumption preferences question was applied. In particular, the binary dependent variable y*i* takes the values “Yes” or “No” and the probability of success *P*(*Y* = *Yes*|*x*) represents the probability that an individual is willing to consume hay milk conditioned by the variables of the questionnaire. It was assumed that consumer obtains maximum utility, if he/she has a positive attitude towards hay milk. 

The probability *P*(*Y* = *Yes*|*x*) of choosing any alternative over not choosing it can be expressed as

*P*(*Y* = *Yes*|*x*) = *Φ* (*xi’β*) (1)

where *Φ* (∙) represents the distribution function of a standard normal random variable [55].

Moreover, the relationship between an independent variable and the outcome (dependent variable) of the probability is interpreted by means of the marginal effects. The marginal effects on dummy variables provide insights into how the explanatory variables shift the probability of frequency of the willingness to consume hay milk.

The marginal effect on dummy variables can be expressed as

Δ = *Φ* (*xi’β*, *d* = *Yes*) − *Φ* (*xi’β*, *d* = *No*) (2)

In particular, using the R software version 3.5.1 [56], the marginal effects were calculated for each variable, holding other variables constant at their sample mean values [55].

## 4. Results

### 4.1. The Sample Characteristics 

The sample was composed of 331 respondents and consisted of 185 females and 146 males with an average age of about 40 years. A slight majority (54.68%) of the respondents had a high educational level (i.e., postgraduate) (Table 1). This results was due to the provenience of sample (Central and Southern Italy), in fact, people comes from Central and South Italy are more educated than North Italian people [57]. Thirty-seven percent of the sample consume semi-skimmed milk or skimmed, 27% of respondents drink high quality pasteurized fresh milk, and 79% of people consume dairy products. 

According to 53% of the sample, milk is a fundamental food in the individual’s diet, and 64% of respondents pay attention to milk taste. 

The willingness to consume hay milk was high. In fact, over 67% of all respondents stated their willingness to consume hay milk, even if 84% of the sample have either never heard about it or consumed hay milk in the past. 

When participants were asked about the associations that came to mind when they heard the term hay milk, their answers differed slightly. In fact, the most frequent association was with cows fed with hay (52% of the respondents), followed by an association with cows fed with hay and meadows (39%). Moreover, associations with livestock management were considered. In particular, many associations with free-ranging (59% of the sample) and free movement of cows and freedom (31%) were drawn. 

In addition, the respondents were asked what milk characteristics could affect their decision to consume hay milk. In fact, people were willing to consume it if it had a different taste to conventional milk (for 58% of the sample), for its nutritional aspects (for 60%), for animal welfare issues (72% of the respondents) and for environmental (67%) and socio-economic (69%) aspects. Finally, according to 65% of the sample, their curiosity could affect respondents’ decision to consume hay milk.

### 4.2. The Probit Model 

In the next step, we applied a probit model for binary choice and calculated the marginal effects. The probit model enables us to successfully and consistently identify the drivers that push the respondents’ willingness to consume hay milk (Mc Fadden Pseudo-R^2^: 0.34); while the marginal effects allow us to understand how each driver shifts the probability (increasing or decreasing) of their willingness to consume hay milk.

**Figure 2:**
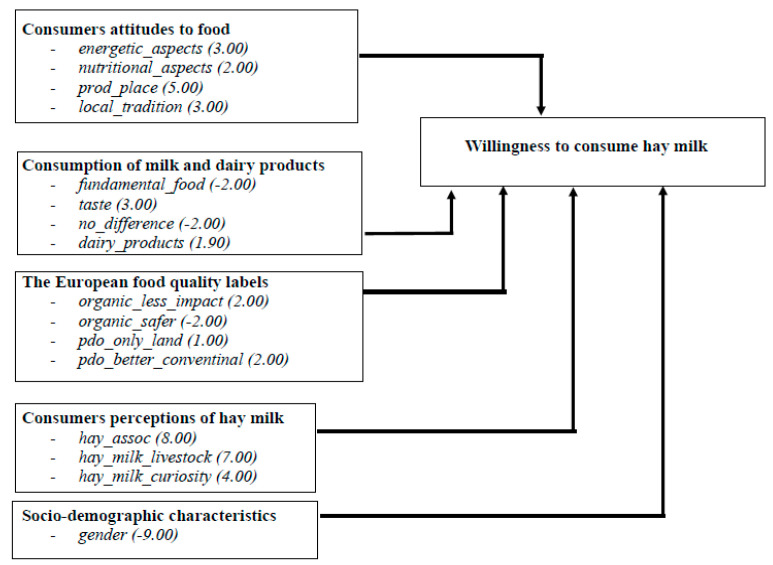
The marginal effects of each variable on willingness to consume hay milk in the conceptual framework.

The results of the binary probit model are showed in Table 2; while Figure 2 shows the marginal effect of each variable (value in parentheses) which belongs to each questionnaire section. 

The findings are very interesting with respect to the understanding of both the perception of hay milk and also the main drivers of this perception (Table 2 and Figure 2). 

In particular, the findings show consumers who pay attention to energetic and nutritional aspects of food are 3-fold and 2-fold more likely to consume hay milk, respectively, than other people.

Furthermore, the production place of food and the local culinary traditions are important aspects to consider in the consumers’ behavior [25,46]. In fact, participants who pay attention to both the production place of food and the local culinary traditions are 5.00 and 3.00 times, respectively, more willing to consume hay milk than other milk consumers. However, those who are willing to consume hay milk do not think milk is a fundamental food in an individual’s diet. 

Moreover, the milk’s taste plays an important role in the willingness to consume hay milk [33]. In fact, those who pay attention to milk’s taste and think that there are differences between them are 3.00 and 2.00 times more willing to drink hay milk than other people. 

Moreover, it is interesting to underline that those who are dairy product consumers are 1.90 times more willing to consume hay milk than other consumers. 

Among the European food quality certification schemes, the organic one is an important driver for the willingness to consume hay milk [33]. In fact, those who believe that organic milk is less impactful than conventional milk but is as safe as conventional milk are more willing to drink hay milk than other respondents. This finding is in line with current literature [58], where it has been shown that consumers perceive differences among unlabeled and labeled milk supplied on the market.

Furthermore, milk with protected designation of origin plays an important role in the willingness to consume milk [50]. In fact, those who believe that PDO products’ characteristics are due to the production place and quality being better than conventional food are 1.00 times and 2.00 times, respectively, more willing to consume hay milk than other people. 

Moreover, those who know the definition of hay milk in terms of feed used and livestock management used are 8.00 times and 7.00 times, respectively, more willing to consume hay milk than other people. This finding underlines that if participants are informed about a specific product, they will show a positive attitude towards the new product [45]. 

Also curiosity about a new product plays an important role in consumer acceptance [45,47]. In fact, those who are curious about hay milk are 4.00 times more likely to consume it than other people. 

Finally, gender is also an important driver in consumer behavior [45]. In fact, females are 9.00 times more likely to willingly consume hay milk than males’ consumers. 

## 5. Discussion

The paper aimed to understand the willingness of 331 people to consume hay milk. In particular, we want to understand which variables drive the people’s willingness to consume hay milk, and if the European food quality certification schemes impact on people’s willingness to consume milk from hay-fed cows.

The sample was composed of a high percentage of females with an average age of about 40 years, and with a higher share of highly educated participants in comparison to the Italian population [33,57]. For these reasons, the findings cannot be interpreted on a Italian national scale as happens in many studies about consumer behavior [25,33,45,47,48]. However, the study showed very interesting results with respect to the understanding of both the perception of hay milk and also the main drivers of this perception. Moreover, we believe that the usefulness of an explorative study (as this) carried out on little-known food issues should not be dismissed so easily.

The willingness of Southern Italians to consume hay milk was high. In fact, over 67% of all respondents stated that they were willing to consume hay milk even if 84% of the sample have either never heard about it or consumed hay milk in the past. Similar results were reached by [52] which showed over 70% of their respondents stating they buy hay milk from South Tyrol.

The results showed that associations with hay milk referred to both cows fed with hay (52% of the sample) and fed with hay and meadow cows (39%); associations with livestock management referred to both free-ranging (59%) and free movement of cows (31% of the sample). Similar results were also shown by [33] which found that people’s associations with hay milk referred to barns and hay (feeding). Moreover, in our case, people’s associations with the term hay milk were important factors in pushing the respondents’ willingness to consume hay milk. In fact, those who knew the definition of hay milk were more likely to consume hay milk than other people. According to the work in [33], potential buyers of hay milk mostly pay attention to cows’ feeding and milk packaging used, while potential buyers of pasture-raised milk pay attention to animal-friendly housing conditions and pasturing for cows. 

In general, quality considerations are important purchasing motivations for people [59] and food choice decisions are based on many aspects, such as people’s egoistic (i.e., health and taste) and altruistic (i.e., environmental and animal welfare issues) motivations.

In particular, according to the work in [33], people evaluate milk types positively according to many aspects such as their healthiness [60] and the sustainability of production. In our case, the nutritional and energetic aspects of food pushed people’s willingness to consume hay milk. Moreover, some studies have shown that concern for animal welfare [61] and environmental issues are identified as major reasons for buying pasture-raised milk [62] as well as the milk types (organic or not) and labels that are perceived and rated differently by people [33,60,63]. In our case, the environmental issues in terms of an organic label were an important driver in the respondents’ willingness to consume hay milk. In fact, according to some authors [58], consumers tend to perceive differences between milk types such as organic and not organic. The differences in production can be claimed through labeling and certification on the product in order to communicate product differentiation to the consumer. According to other authors [33], product differentiation could be of particular economic interest for farmers that can use their land in a way that is supported by society, for example, through maintaining traditional landscapes with small-scale farms. Some authors [64] showed that quality labels, e.g., the label for food with PDO could reduce the risk of small farms being abandoned. In this framework, the findings are much more interesting if we think that the European food quality certification schemes such as origin (i.e., PDO) and organic labels can be seen as independent extrinsic cues to the consumers and lead to certain product preference according to their quality and perceptions. In fact, participants who pay attention to both the production place of food, the local culinary traditions and believe PDO products characteristics are due to the production place and that its quality is better than conventional food are more likely to willingly consume hay milk than other consumers. According to some authors [31,40], European consumers are showing renewed interest in traditional food and this growing interest in quality and traditional products generates a demand for agricultural products with specific, identifiable characteristics, particularly those that are linked to their geographical origin and their production method [27].

Furthermore, the milk’s taste is an important aspect in the consumers’ willingness to consume hay milk [33]. Some authors [65] showed that the purchasing interest for organic milk was pushed by the qualities of fresh and aromatic taste, safety, high-quality, healthy, and high nutritional aspects of milk. According to the authors of [62], many of these aspects can be found in pasture-raised milk. Furthermore, in our case, the milk’s taste drives people’s willingness to consume hay milk. 

Moreover, some authors [49] found milk consumers are also cheese consumers, and in our case, those who were dairy product consumers were more likely to willingly consume hay milk than other consumers.

As far as gender influence is concerned, it is interesting to note that the empirical results reported in Table 2 and Figure 2 showed that women are on average more prone to drink hay milk than men, confirming part of the current literature about gender influence on consumer behavior [45].

Finally, as happens in many studies about consumer behavior (see, e.g., in [45]) curiosity about a new product is an important factor that drives people’s willingness to consume it. In fact, in our case, curious people are more willing to consume hay milk than other consumers. 

## 6. Conclusions

In the last few years, it has become increasingly important to the food sector to understand consumers’ behavior when these consumers are confronted with more niche labels. Consumers’ appreciation is crucial for the success of a new food on the market.

During the last years, Italian consumers showed a decrease in the consumption of meat, milk, and dairy products [66]. By contrast, milk consumption is undergoing an evolution with a growth of so-called green products [67,68]. In general, consumers’ interest for organic and local production has grown tremendously [69].

In this context, a reflection on the marketing strategies of dairy companies and the opportunities for farms in marginalized areas are appropriate. 

Although the sample of this research cannot be considered representative of the entire Italian population, the results obtained gave interesting hints to understand the process of consumer decision-making. In fact, further studies should be necessary to better understand the Italian consumers’ propensity towards hay milk acceptance, in terms of their individual preferences and attitudes. However, our findings showed a strong interest in respondents towards hay milk and highlighted some important marketing implications for the Italian milk sector. In fact, the findings highlighted the European food quality certification schemes such as origin (i.e., PDO) and organic labels can be seen as extrinsic cues to the consumers and lead to certain product preference according to their quality and perceptions.

Profiling consumers who are willing to consume hay milk could be a first step towards a better understanding of consumers’ decisions on labeled food. Our results indicated that the European food quality certification schemes such as origin (i.e., PDO) and organic labels are two important factors that could drive people’s willingness to consume hay milk. 

The hay milk consumer in our survey could be described as a curious female, who consumes semi-skimmed milk, pays attention to both the production place of food and the local culinary traditions, and is more label-conscious and environmentally friendly. These findings might be useful insights for farmers that focus on product differentiation to survive on the market. In other words, the study findings could encourage discussion about a niche market to boost local growth, initiating a process of improving livelihoods, certification of products and use of the marketing tools addressed towards a specific milk consumer’s profile.

However, the study shows some limitations that could be considered in future research. In fact, these limitations are given both the recruitment method used (and thus the sample cannot be considered representative of the entire Italian population as mentioned above) and, in the survey, in which verbal descriptors have been used to identify the European food quality certification schemes, which might mimic a real market in a less realistic way. To avoid this limitation, further research should simulate real shopping environments where the choice sets are designed with visual labeling elements, such as European labels image to increase the accuracy of the results. Moreover, further research should be focused on the people’s willingness to pay for hay milk; later, it could be interesting understand if the individuated price is profitable for farmers to offer the opportunity to add some new insights and to propose further discussion on a new niche market as hay milk one.

## Figures and Tables

**Table 1:** Socio-demographic characteristics of the sample (*n* = 331).

Variables	%
Gender	
Male	44.11
Female	55.89
Total	100.00
Education	
Low_education	45.32
High_education	54.68
Total	100.00

Source: Our elaboration on survey data.

**Table 2:** Estimates of the binary probit model (*n* = 331).

Variables	*β*	Standard Error	z-Value	*p*-Value	Marginal Effects
Intercept	−2.67	0.59	−4.49	<0.000	−0.63
*energetic_aspects*	0.11	0.06	1.86	<0.05	3.00
*nutritional_aspects*	0.12	0.07	1.82	<0.05	2.00
*origin_material_raw*	−0.14	0.08	−1.59	n.s.	-
*prod_place*	0.22	0.09	2.34	<0.01	5.00
*local_tradition*	0.11	0.06	−1.87	<0.05	3.00
*fundamental_food*	−0.08	0.04	−1.80	<0.05	−2.00
*taste*	0.12	0.04	2.55	<0.01	3.00
*no_difference*	−0.09	0.03	−2.76	<0.001	−2.00
*dairy_products*	0.82	0.18	4.51	<0.000	1.90
*organic_less_impact*	0.09	0.05	1.82	<0.05	2.00
*organic_safer*	−0.09	0.04	−2.00	<0.01	−2.00
*pdo_only_land*	0.06	0.03	1.44	<0.01	1.00
*pdo_better_conventional*	0.09	0.05	2.07	<0.01	2.00
*hay_milk*	0.52	0.28	1.88	n.s.	-
*hay_milk_past*	5.27	0.21	0.02	n.s.	-
*hay_assoc*	0.33	0.15	2.18	<0.01	8.00
*hay_milk_livestock*	0.29	0.13	2.19	<0.01	7.00
*hay_milk_curiosity*	0.16	0.03	4.56	<0.000	4.00
*hay_milk_less_env_impact*	0.08	0.04	1.92	n.s	-
*gender*	−0.36	0.18	−2.03	<0.01	−9.00
AIC: 319.45 Mc Fadden Pseudo-R^2^: 0.34					

Note: n.s. means variable with not significant value. Source: Our elaboration on survey data.
